# Effect of vascular endothelial growth factor rs35569394 in esophageal cancer and response to chemotherapy

**DOI:** 10.17305/bjbms.2021.5891

**Published:** 2023-03-16

**Authors:** Zishan Wang, Chenwei Li, Xinjian Li, Jianguang Shi, Weijie Wu

**Affiliations:** 1 Department of Thoracic Surgery, Ningbo First Hospital, Ningbo Hospital, Zhejiang University School of Medicine, Ningbo, Zhejiang, China

**Keywords:** Genetic polymorphism, esophagus cancer, vascular endothelial growth factor (VEGF), angiogenesis, rs35569394, chemotherapy

## Abstract

The objective of this study was to investigate the possible association between the single-nucleotide polymorphism, rs35569394, of the vascular endothelial growth factor (*VEGF*) gene and the risk of esophageal cancer (EC) in the Han Chinese population. A total of 290 EC subjects and 322 ethnically matched unrelated healthy controls free from the esophageal disease were studied. Genomic DNA was isolated from peripheral blood by salting out. Genotyping of *VEGF* rs35569394 polymorphism was carried out through polymerase chain reaction followed by agarose gel electrophoresis. The results showed that the distribution of genotypes was significantly different across the gender groups (*p* ═ 0.032) and clinical stages of the esophageal cancer (*p* ═ 0.034). *VEGF* rs35569394 was associated with EC risk (*p* ═ 0.012, OR ═ 1.34). A gender analysis breakdown showed that rs35569394-D allele frequency was significantly higher in females than in the controls (*p* ═ 0.0004, OR ═ 1.81). Moreover, significant associations were also found in females under the dominant model (II vs. ID+DD: χ^2^ ═ 8.18, *p* ═ 0.003, OR ═ 2.12) and the recessive model (II+ID vs. DD: χ^2^ ═ 8.25, *p* ═ 0.004, OR ═ 2.39). In addition, we found that the genotype, rs35569394-DD, was associated with a complete response and partial response to chemotherapy when compared with rs35569394-II (χ^2^ ═ 4.67, *p* ═ 0.030, OR ═ 0.47). In conclusion, our case–control study showed that the *VEGF* rs35569394 was significantly associated with the clinical stages and the increased risk of EC in Han Chinese females. In addition, the genotype rs35569394-DD showed a better response to chemotherapy.

## Introduction

Esophageal cancer (EC) is one of the most common malignancies. The worldwide incidence of EC is 5.9 per 100,000 population, and the mortality is 5.5 per 100,000 [1]. EC is considered among the four leading causes of cancer-related mortality in China, although both the incidence and the mortality rate associated with EC have been declining in recent years [2]. EC is a complex disease, involving a variety of environmental and genetic factors. Numerous epidemiological studies have indicated that many environmental risk factors, such as age [3], genetic factors [4], unhealthy eating habits [5], smoking tobacco, and alcohol consumption [6], are associated with EC. In the case of genetic factors, genome-wide association studies have identified many single-nucleotide polymorphisms (SNPs) associated with EC in different populations [7, 8].

Angiogenesis plays a critical role in tumor dissemination, invasion, and metastasis [9]. Its complex course is regulated by various growth factors, among which, the vascular endothelial growth factor (*VEGF*) plays an important role [10]. Human *VEGF* (OMIM 192240) is located on chromosome 6p21.3. Gene expression is correlated with the degree of vascularization and a poor prognosis in many malignancies, including carcinoma of the brain, cervix, bladder, gastric, esophagus, colon, breast, kidney, ovary, as well as in soft-tissue sarcomas and pediatric tumors [11]. Variations in the DNA sequence of *VEGF* may alter the production and/or activity of *VEGF*, thereby causing interindividual differences in the development and progression of tumors [12].

The insertion/deletion (I/D) polymorphism (rs35569394), which is in the promoter region of *VEGF* at position – 2549, relative to the translation start site, has been linked to altered transcriptional activity [13]. Several studies have analyzed rs35569394 polymorphism in the context of susceptibility to malignancies in different populations, but the results were inconsistent. Wafi et al. [14] showed that the haplotype of *VEGF* rs35569394 and rs699947 was significantly associated with an increased risk for urothelial bladder cancer in the Tunisian population. Rezaei et al. [15] observed that *VEGF* rs699947 was a risk factor for breast cancer development in an Iranian population, although no significant association was found with *VEGF* rs35569394. However, He et al. [16] were unable to detect a relationship between *VEGF* rs35569394 and hepatocellular carcinoma at either the allele or the genotype level, in a Chinese population. Cancer has been suggested to have different genetic predictors [17], incidence, and survival among different ethnic populations [18]. Therefore, it is necessary to investigate whether *VEGF* rs35569394 is a genetic factor responsible for EC in the Chinese population.

To the best of our knowledge, there are currently no published reports regarding *VEGF* rs35569394 polymorphism with respect to EC. In this study, we recruited 290 EC patients and 322 normal individuals originating from Ningbo city in East China and performed a case–control test to investigate the association between *VEGF* rs35569394 polymorphism and severity of EC in homogenous samples. We also investigated the role played by this SNP in the response to chemotherapy in the patients.

## Materials and methods

### Subjects

The current retrospective case–control study consisted of 290 newly diagnosed patients with histopathologically confirmed EC from the Department of Cardiothoracic Surgery, the Ningbo First Hospital, between October 2012 and May 2021. As well, 322 cancer-free control subjects were recruited randomly from a similar geographical region and were genetically unrelated to the cases. Each subject was interviewed using a questionnaire to obtain information on epidemiological factors such as dietary habits, smoking, alcohol consumption, and family history, among others. In accordance with the principles of the Helsinki Declaration, written informed consent was obtained from all subjects before inclusion in the study. The study protocol was approved by our Institutional Research Ethics Committee.

**Figure 1.**
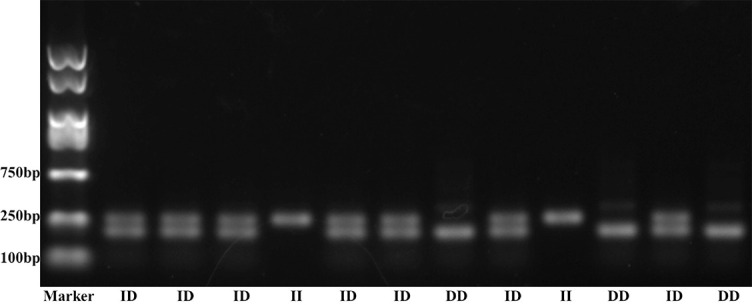
**Polymerase chain reaction (PCR) amplification products for *VEGF* I/D –2549 gene polymorphism.** Marker: PCR marker of 2000 bp; II: Wild type; ID: Heterozygous mutation; DD: Homozygous mutation.

### Chemotherapy

Patients were treated with the following modified [19] first-line treatment: Oxaliplatin, 85 mg/m^2^ on day 1 (Jiangsu Hengrui Medicine Co., Ltd., Lianyungang, China); calcium folinate, 400 mg/m^2^ on days 1-5 (Jiangsu Hengrui Medicine Co., Ltd.) combined with 5-fluorouracil, 400 mg/m^2^ on days 1-5 (Tianjin Jinyao Amino Acids Co., Ltd., Tianjin, China). The regimen was repeated every 3 weeks. This regimen was administered until either disease progression was controlled, or unacceptable levels of toxicity occurred, or the patient refused treatment.

Clinical response was evaluated every two cycles through computed tomography. The disease control rate (complete response [CR] + partial response [PR] + stable disease [SD] + progressive disease [PD]) was evaluated according to the Response Evaluation Criteria in Solid Tumors guidelines [20]. Chemotherapy was stopped in case of disease progression, patient refusal, or unacceptable toxicity.

### SNP genotyping

A 5 ml venous blood sample from each subject was collected in an EDTA coated Vacutainer and stored at −20^∘^C. Total genomic DNA was isolated from peripheral blood leukocytes through the salting out procedure [21]. *VEGF* polymorphism rs35569394 primers were from the published paper [22, 23]. Polymerase chain reaction (PCR) amplification was performed using the following conditions: Initial denaturation at 95^∘^C for 10 min followed by 30 cycles with denaturation at 94^∘^C for 1 min, annealing at 57^∘^C for 1.5 min, extension at 72^∘^C for 2 min, and a final extension at 72^∘^C for 10 min. PCR products were separated using electrophoresis through 3% agarose gel stained with ethidium bromide. Different genotypes were indicated by the presence of different bands: 229 bp (I allele with the 18 bp insertion) and 211 bp (D allele with no insertion) (Figure 1).

### Statistical analysis

Genotype distribution was analyzed using Pearson’s Chi-square test and the Hardy–Weinberg equilibrium (HWE). Discrete data were compared using Pearson’s Chi-square test or Fisher’s exact test. Quantitative data were compared using one-way analysis of variance or the Kruskal–Wallis test. All data were analyzed with SPSS statistical software (version 16.0). Statistical significance was set at a two-tailed *p* < 0.05.

## Results

### Characteristics of the study population

Comparison of clinical characteristics in the three genotypes is shown (Table 1). The case group consisted of 290 EC patients (153 males and 137 females) with a mean age of 62.1 (±7.34) years. The control group consisted of 322 cancer-free subjects (173 males and 149 females) with a mean age of 60.9 (±9.11) years. Significant differences were found in the gender (*p* ═ 0.032) and clinical stage (*p* ═ 0.033) among the three genotypes. However, no significant differences were found in the tumor grade, tumor location, histology, or the carcinoembryonic antigen (*p* > 0.05).

**Table 1:** Clinical characteristics and *VEGF* rs35569394 genotype distribution in case group*

**Characteristics**	**Genotype (n)**			***p*-value**
	**II**	**ID**	**DD**	
All	77	154	59	
Age				
≤59	34	68	30	0.979
>59	43	86	29	
Gender				
Male	46	85	22	**0.032**
Female	31	69	37	
Tumor grade				
Well (G1)	11	18	9	0.627
Moderate (G2)	15	49	23	
Poorly (G3)	51	87	27	
Tumor location				
Upper third	16	26	10	0.768
Middle third	28	50	23	
Lower third	33	78	26	
Tumor histology				
Adenocarcinoma	4	12	5	0.763
Squamous cell carcinoma	73	142	54	
Clinical stage				
I	15	29	6	**0.033**
II	27	58	15	
III	26	47	20	
IV	9	20	18	
Carcinoembryonic antigen				
<5 ng/mL	42	82	30	0.623
>5 ng/mL	35	72	29	

*The case group consisted of 290 EC patients (153 males and 137 females) with a mean age of 62.1 ± 7.34 years. The control group consisted of 322 cancer-free subjects (173 males and 149 females) with a mean age of 60.9 ± 9.11 years. VEGF: Vascular endothelial growth factor; EC: Esophageal cancer; II: Wild type; ID: Heterozygous mutation; DD: Homozygous mutation.

### Genotype analysis

Genotypic and allelic comparison between ECs and controls for *VEGF* rs35569394 is shown (Table 2). Departure from HWE was not observed for rs35569394 in the controls (*p* > 0.05). The results indicated that SNP rs35569394 was associated with a risk for EC (genotype, χ^2^ ═ 6.96, *p* ═ 0.031, OR ═ 1.27, 95% CI ═ 1.08−1.74; allele, χ^2^ ═ 6.35, *p* ═ 0.012, OR ═ 1.34, 95% CI ═ 1.07−1.68). Furthermore, when the data analysis was stratified according to gender group with respect to allele and genotype frequencies, significant differences were evident in female ECs both at the genotype level (χ^2^ ═ 12.33, df ═ 2, *p* ═ 0.002, OR ═ 1.84, 95% CI ═ 1.30−2.59) and the allele level (χ^2^ ═ 12.71, *p* ═ 0.0004, OR ═ 1.81, 95% CI ═ 1.30−2.53).

**Table 2:** Distribution of genotypes and alleles in both groups

**SNP/Gender**	**Group**	**Genotype (n)**	**χ^2^**	***p* (d.f.=2)**	**OR (95%CI)**	**Allele (n)**	**χ^2^**	***p* (d.f.=1)**	**OR (95%CI)**
rs35569394		II/ID/DD				I/D			
All	Case	77/154/59			1.27	308/272			1.34
	Control	110/168/44	6.96	**0.031**	(1.08–1.74)	388/256	6.35	**0.012**	(1.07–1.68)
Male	Case	46/85/22			1.03	177/129			1.03
	Control	53/96/24	0.047	0.974	(0.73–1.44)	202/144	0.04	0.841	(0.77–1.41)
Female	Case	31/69/37			1.84	131/143			1.81
	Control	57/72/20	12.33	**0.002**	(1.30–2.59)	186/112	12.71	**0.0004**	(1.30–2.53)

Bold: *p* < 0.05.

Positive associations were observed between rs35569394 and EC under the dominant model (II vs. ID+DD: χ^2^ ═ 4.16, *p* ═ 0.041, OR ═ 1.43, 95% CI ═ 1.01−2.03) and recessive model (II+ID vs. DD: χ^2^ ═ 4.86, *p* ═ 0.024, OR ═ 1.61, 95% CI ═ 1.05−2.47) (Table 3). In the gender analysis, significant results were found in both the dominant model (χ^2^ ═ 8.18, *p* ═ 0.003, OR ═ 2.12, 95% CI ═ 1.26−3.56) and the recessive model in females (χ^2^ ═ 8.25, *p* ═ 0.004, OR ═ 2.39, 95% CI ═ 1.31−4.36). However, no significant differences were observed between cases and controls in the male group (*p* > 0.05).

**Table 3:** Comparison of the dominant model and recessive model in rs35569394 between cases and controls by gender

**SNP/Gender**	**Group**	**Dominant model**		**χ^2^**	***p***	**OR (95%CI)**	**Recessive model**		**χ^2^**	***p***	**OR (95%CI)**
rs35569394		II	ID+DD				II+ID	DD			
All	Case	77	213				231	59			
	Control	110	212	4.16	**0.041**	1.43 (1.01–2.03)	278	44	4.86	**0.024**	1.61 (1.05–2.47)
Male	Case	46	107				131	22			
	Control	53	120	0.01	0.92	1.03 (0.64–1.65)	149	24	0.53	0.47	1.26 (0.67–2.37)
Female	Case	31	106				100	37			
	Control	57	92	8.18	**0.003**	2.12 (1.26–3.56)	129	20	8.25	**0.004**	2.39 (1.31–4.36)

Bold: *p* < 0.05.

### *VEGF* rs35569394 and chemotherapy efficacy

A total of 290 subjects were evaluated for response. The overall response rate was 63.1%, with 26 CR and 157 PR; while 65 cases had SD and the remaining 42 cases had PD. Correlation between *VEGF* rs35569394 and response to chemotherapy is shown (Table 4). Logistic regression analysis, indicated that the genotype, rs35569394-DD, was associated with a CR+PR to chemotherapy when compared with rs35569394-II (χ^2^ ═ 4.67, *p* ═ 0.030, OR ═ 0.47, 95% CI ═ 0.24−0.94).

**Table 4:** Association between *VEGF* rs35569394 and clinical response to chemotherapy

**Genotype (n)**		**CR+PR**	**SD+PD**	**χ^2^**	***p***	**OR (95%CI)**
II	77	47	30			1 (Ref)
ID	154	101	53	2.89	0.089	1.65 (0.92–2.94)
DD	59	25	34	4.67	**0.030**	0.47 (0.24–0.94)

Bold: *p* < 0.05; CR: Complete response; PR: Partial response; SD: Stable disease; PD: Progressive disease.

## Discussion

In the current case–control study, 290 EC patients and 322 unrelated healthy control individuals were analyzed. Our results indicated that *VEGF* rs35569394 was strongly associated with the clinical stages and the risk of EC in Han Chinese. In addition, the gender-stratified comparison showed that rs35569394 was associated with EC risk in females. Patients with rs35569394-DD also showed a better response to chemotherapy.

*VEGF* polymorphisms were significantly associated with the development of EC in both Asian and Caucasian populations [24, 25]. The haplotypes of *VEGF* polymorphisms could increase the risk of EC in a Han Chinese population [26]. Bradbury et al. [27] suggested that *VEGF* polymorphism rs3025039 could improve the overall survival (OS) of EC and the CGC haplotype of three *VEGF* SNPs (rs833061, rs2010963, and rs3025039) could reduce OS compared with all other patients. *VEGF* rs35569394 polymorphism has previously been associated with a risk for different malignancies. Kapahi et al. [28] reported that serum *VEGF*-C levels were considerably higher in breast cancer patients, the distribution of rs35569394 genotypes was significantly different in the clinical stages and that *VEGF* rs35569394-D plays a role in susceptibility to breast cancer in the North Indian population. Bruyere et al. [29] suggested that rs35569394-I allele was significantly associated with an increased risk for developing renal cell carcinoma in the French population. However, He et al. [16] were unable to find an association between rs35569394 and hepatocellular carcinoma for either the genotype or the allele in the Chinese population. The results of the present study suggested that the *VEGF* rs35569394 genotype is strongly associated with the severity of EC, *VEGF* rs35569394-D is associated with increased susceptibility to EC in Han Chinese females, and thereby shows potential as a biomarker of the clinical outcome of EC in our population.

Gender-specific differences have been reported among many genetic variations, including in *VEGF*. The previous studies suggested that *VEGF* rs699947 polymorphism was associated with an increased risk of osteosarcoma [30] and that the genotype of rs699947-AA was associated with an increased risk of osteosarcoma in young males with a family history of cancer [31]. Lysogorskaia et al. reported that *VEGF* rs699947 was most associated with the risk of amyotrophic lateral sclerosis in the male group [32]. Furthermore, rs35569394 polymorphism has been reported to be in perfect linkage disequilibrium with rs699947 polymorphism, where individuals with the rs699947-A allele exhibit an 18 bp insertion (rs35569394-I), while those with the rs699947-C allele showed a deletion (rs35569394-D) [33, 34]. In our study, the results indicated that rs35569394 was associated with EC risk only in females. This suggested that the role played by *VEGF* rs35569394 in EC pathogenesis may be gender specific.

*VEGF* is a potent and critical mediator of physiological as well as pathological vasculogenesis and angiogenesis in tumor growth. In chemotherapy, *VEGF* expression is significantly associated with therapeutic effects. Evidently, *VEGF* production may be partially subject to genetic control. Saleh et al. indicated that genetic variants of *NOS3*, *CD14*, *MMP3,* and *IL4R* were implicated in the determination of *VEGF* expression and plasma levels [35]. *VEGF* polymorphism also showed a significant association with malignancy [36]. Bhaskari et al. observed that different haplotypes may enhance *VEGF* expression in both plasma and tissues, which was correlated with a poor prognosis as well as recurrence in epithelial ovarian cancers [36]. *In vitro* function studies conducted by Cooper et al., showed that the presence of rs35569394-I allele in the promoter region could lead to enhanced expression of *VEGF* [37]. Another study found that the genotype rs35569394-IIdisplayed a 1.95-fold increase in transcriptional activity compared with that of rs35569394-DD [13]. Our findings indicated that the rs35569394 genotypes were significantly associated with response to chemotherapy by EC and that rs35569394-DD produced a better response to chemotherapy. This may provide a possible explanation for the higher response rates to chemotherapy observed in EC patients carrying the rs35569394-DD genotype, which may be due to decreased transcription.

Some limitations were associated with the current study. First, only one SNP was checked for its relationship with EC, other polymorphisms of *VEGF* may also contribute to the association. Second, the sample size of patients with EC was relatively small, which may have limited the statistical power of tests conducted to find the difference between groups. Therefore, further studies utilizing larger sample sizes may be needed to confirm these results.

## Conclusion

Our case–control study demonstrated that *VEGF* polymorphism rs35569394 was significantly associated with the clinical stages and the increased risk of EC in Han Chinese females. In addition, those carrying the genotype rs35569394-DD showed a better response to chemotherapy.
